# Ocular and confocal manifestations of Mainland Chinese with Fabry disease: a cross-sectional controlled study

**DOI:** 10.1186/s13023-025-03940-9

**Published:** 2025-08-10

**Authors:** Yao Xu, Yingjie Chen, Jiali Fan, Sasa Kou, Xinyu Zhuang, Bingyuan Zhou, Xiaofeng Zhang

**Affiliations:** 1 Department of Ophthalmology, The Fourth Affiliated Hospital of Soochow University, No.9,Chongwen Road, Suzhou, 215000 China; 2 Department of Cardiology Echocardiography, The First Affiliated Hospital of Soochow University, No. 188, Shizi Street, Suzhou, 215000 China; 3 Department of Ophthalmology, Suzhou Vocational Health College, Suzhou, Jiangsu China

**Keywords:** Fabry disease, Ocular manifestations, In vivo confocal microscopy, Verticillata opacity, Enzyme replacement therapy

## Abstract

**Background:**

This cross-sectional controlled study aims to characterize ocular manifestations and corneal microstructure via in vivo confocal microscopy (IVCM) in mainland Chinese patients with Fabry disease (FD). We evaluated 30 FD patients (mean age: 38 ± 14.41 years; range: 10–60 years), divided equally into enzyme replacement therapy (ERT)-treated and untreated groups, alongside 30 age- and gender-matched healthy controls. Slit-lamp examinations assessed ocular manifestations, while IVCM was employed to analyze corneal microstructure.

**Results:**

Eighteen FD patients presented with corneal verticillata (CV) opacities. High-reflective intracellular inclusions were identified in the corneal basal epithelial cells in the majority of FD patients (22 out of 30). IVCM detected increased dendritic cells (DCs) in three FD patients. The nerve fiber layer showed an increased corneal nerve tortuosity coefficient (*P* < 0.001), decreased nerve fiber density (NFD) (*P* = 0.033), decreased nerve fiber length (NFL) (*P* = 0.012), and reduced fractal dimension (*P* = 0.010) in FD patients compared to healthy controls. Reduced transparency of the anterior corneal stroma and the presence of visible microdots were observed in 11 out of 30 FD patients. Endothelial morphological parameters in FD patients showed no obvious differences compared to healthy controls. α-galactosidase A (α-Gal A) activity was negatively correlated with Mainz Severity Score Index (MSSI) scores (*P* = 0.001), whereas plasma globotriaosylsphingosine (lyso-Gb3) levels and posterior capsular opacification exhibited a direct correlation with MSSI scores(*P* = 0.002). None of these changes showed significant differences in FD patients, regardless of ERT.

**Conclusions:**

This study substantially enhances our understanding of FD-associated ocular alterations in the mainland Chinese demographic. The presence of CV opacities, posterior capsular opacification, or distinct changes observed in IVCM offers the potential for early detection of FD. Additionally, there is a notable increase in DCs and a positive correlation between posterior capsular opacification and MSSI scores. These findings support the integration of ocular biomarker screening into standardized FD diagnostic protocols to facilitate pre-symptomatic interventions, particularly in familial risk cohorts.

## Background

Fabry Disease (FD) is an X-linked lysosomal storage disorder caused by mutations in the GLA gene (Xq22.1), encoding α-galactosidase A (α-Gal A). This enzyme catalyzes degradation of globotriaosylceramide (GL-3) and its byproduct globotriaosylsphingosine (lyso-Gb3). Deficiency or loss of α-Gal A activity leads to GL-3 accumulation in the lysosomes of endothelial cells, renal tubules, neurons, and cardiac tissue, disrupting cellular function and contributing to multi-organ manifestations such as neuropathy, cardiomyopathy, renal failure, and gastrointestinal disturbances [1].

Over 1,100 GLA mutations have been identified, with 60% being missense variants [2]. These mutations result in phenotypic diversity, ranging from the classic early-onset form (Type 1) to the late-onset nonclassic form (Type 2). Classic FD presents in childhood (ages 4–8) with severe manifestations such as angiokeratomas, acroparesthesia, and hypohidrosis, while late-onset forms typically show predominant cardiac or renal involvement with symptoms appearing in adulthood [3]. Ocular findings, such as corneal verticillata (CV) and retinal vascular tortuosity, are characteristic and serve as valuable diagnostic markers.

Phenotypic variability is influenced not only by GLA gene mutations but also by residual enzyme activity and X-inactivation patterns in females. Males, with only one X chromosome, are usually more severely affected. In contrast, females may have milder symptoms due to skewed X-inactivation, though approximately 20–30% of females with a GLA mutation exhibit significant organ involvement [3]. The α-Gal A enzyme’s role in degrading GL-3 is crucial for cellular homeostasis. Mutations often result in misfolded proteins or decreased enzyme stability, leading to GL-3 accumulation and subsequent organ damage [4, 5].

Additionally, the disruption of α-Gal A function influences cellular processes such as autophagy, increasing lysosomal storage dysfunction and cellular stress. This contributes to the multi-system nature of FD, affecting not only the heart, kidneys, and nervous system, but also autonomic function, causing neurological symptoms like sensorineural hearing loss and sweating abnormalities [1, 6–9].

Diagnosis relies on triad confirmation: reduced α-Gal A activity, genetic identification of GLA variants, and elevated plasma lyso-Gb3. Histopathological evidence of lysosomal GL-3 deposits in biopsies provides additional diagnostic support. Comprehensive evaluation is essential due to the systemic involvement of FD, impacting cardiovascular, cerebrovascular, renal, and ocular systems [8, 10].

Current therapies focus on addressing enzymatic deficiency, reducing pathological substrate accumulation, and managing multisystem complications. Enzyme replacement therapy (ERT) [11, 12], introduced in 2001, remains the cornerstone of treatment, with recombinant α-Gal A used to degrade lyso-Gb3 and prevent further organ damage. Two formulations, agalsidase alfa and beta, are administered via biweekly, demonstrating efficacy in stabilizing renal function and reducing cardiac hypertrophy [13, 14]. For patients with amenable GLA mutations, oral chaperone therapy (e.g., migalastat) enhances enzyme stability, though it is mutation-specific. Emerging therapies under investigation include PEGylated ERT formulations, gene therapy using adeno-associated virus (AAV), and substrate reduction agents targeting glycosphingolipid synthesis [12].

Symptomatic management for neuropathic pain, autonomic dysfunction, and cardiovascular complications includes gabapentinoids, ACE inhibitors, and implantable cardiac devices. Multidisciplinary care with regular monitoring through advanced imaging and biomarker tracking has shown to improve life expectancy and quality of life [12].

Ophthalmologic manifestations are often among the first signs of FD, typically appearing in the second decade of life. While relatives contribute to about half of diagnoses, specialists such as nephrologists, neurologists, and cardiologist are more frequently involved in diagnosis, with ophthalmologists playing a minor role. Enhancing ophthalmologists’ recognition of these ocular signs is crucial for early diagnosis [15]. CV is a common ocular symptom, appearing as whorl-like opacities in the corneal epithelial and subepithelial layers [16]. Tortuous conjunctival and retinal vessels and posterior lens opacities, are commonly identified in FD patients, collectively recognized as Fabry-related ocular lesions [1]. Although these lesions typically do not impair vision, they often serve as early and distinctive indicators of the disease [9].

This study aims to characterize ocular manifestations in FD patients, focusing on microstructural changes in various corneal layers using in vivo confocal microscopy (IVCM). It is the first study conducted in mainland China to investigate alterations in the corneal nerve fiber layer in FD patients, and its findings will enhance diagnostic precision and contribute to a better understanding of FD-related ocular changes.

## Method

### Study design

Cross-sectional controlled study. Patients from the FD cohort and healthy controls were recruited from the Department of Ophthalmology at the Fourth Affiliated Hospital of Soochow University during the period from August 2022 to August 2023. The study adhered to the principles of the Helsinki Declaration, with informed consent obtained from all patients and approval from the Clinical Research Ethics Committee of the Fourth Affiliated Hospital of Soochow University.

### Patient**s**

FD diagnosis was confirmed based on a comprehensive clinical assessment and laboratory tests, including GLA gene analysis, α-Gal A activity, and lyso-Gb3 levels. Detailed medication profiles were documented for FD patients undergoing ERT. To assess the systemic involvement, a thorough examination was conducted, including the review of medical and family histories, physical and cardiac assessments (e.g., electrocardiogram, echocardiography, and magnetic resonance imaging [MRI]), and evaluation of renal function (kidney biopsy was performed when clinically indicated). The severity of general, neurological, cardiovascular, and renal symptoms was quantified using the Mainz Severity Score Index (MSSI), with scores categorized as mild (< 20), moderate (20–40), or severe (> 40).

Exclusion criteria for the FD group included: (1) systemic autoimmune diseases other than FD, (2) history of ocular trauma or surgery, (3) any corneal pathology, (4) use of contact lenses, (5) incomplete medical records. Healthy volunteers without ocular or systemic diseases comprised the control group, excluding those who used contact lenses or had undergone refractive surgery.

### Study procedures

Each patient underwent a comprehensive ophthalmological assessment, which included slit-lamp examination and corneal imaging by a single expert ophthalmologist to assess for CV opacities. To minimize inter-examiner variability and reduce potential biases, IVCM was performed by a separate, independent expert examiner on the same day. This double-blind approach ensured that neither examiner was aware of the findings of the other.

The Heidelberg Retinal Tomograph III-Rostock Cornea Module (Heidelberg Engineering GmbH, Heidelberg, Germany) was used to perform laser scanning confocal microscopy on all patients. The standardized imaging protocol involved selecting three images from each distinct corneal layer (epithelial, nerve fiber, stromal, and endothelial) at consistent depths for each patient’s examination. For each corneal layer, the following parameters were measured:

Epithelial Layer: Cell area, density, circularity, major axis, minor axis, mean cell size, light density.

Nerve Fiber Layer: Nerve fiber density (NFD), nerve branch density (NBD), nerve fiber length (NFL), nerve fiber area (NFA), nerve fiber width (NFW), fractal dimension, and tortuosity coefficient.

Stromal Layer: Cell area, density, circularity, major axis, minor axis, mean cell size, light density.

Endothelial Layer: Cell area, density, circularity, major axis, minor axis, mean cell size, light density.

Quantification of nerve fiber parameters was performed using ACCmetrics version 3.0 and CCmetrics version 1.1 (University of Manchester, UK). These software tools enabled accurate measurement and analysis of nerve fiber morphology and structure. For the epithelial, stromal, and endothelial layers, ImageJ software was used to quantify the parameters mentioned above. To ensure consistency and accuracy, the median values from the three selected images of each layer were used for further analysis.

### Statistical analysis

Statistical analyses were conducted using SPSS version 25.0 (Chicago, IL, USA). Descriptive statistics for continuous variables were presented as mean ± standard deviation (SD) or median (interquartile range [IQR]) depending on the distribution of the data. Categorical variables were analyzed using the Pearson χ² test or Fisher’s exact test, and normality of continuous variables was assessed using the Kolmogorov-Smirnov test. For normally distributed data, group comparisons were made using the independent samples t-test, while for non-normally distributed data, the Mann-Whitney U test was applied. Spearman’s correlation coefficient was used to examine the relationships between disease severity scores and both corneal morphometric parameters and enzyme activity. Statistical significance was set at *p* < 0.05 for all tests, which were two-tailed.

## Results

Data were collected from the right eyes of 30 FD patients, each with comprehensive medical profile, and 30 age- and gender-matched healthy controls. All 30 FD patients were referred to our department by the Department of Cardiology Echocardiography at the First Affiliated Hospital of Soochow University. Thirteen patients were diagnosed through genetic screening following confirmation of FD in family members. Sixteen patients initially manifested characteristic clinical features including hypertrophic cardiomyopathy, renal insufficiency, acroparesthesia, hypohidrosis, auditory decline, and visual deterioration, with subsequent confirmation of FD through comprehensive diagnostic evaluations. One additional case was incidentally identified as FD during routine health examination.

Patient demographics are summarized in Table 1. The FD group consisted of 11 males and 19 females; with a mean age of 38 *± 14.41 years (range: 10–60).* Among these, 15 (7 males, 8 females) were undergoing ERT, as detailed in Table 2, while the remaining 15 (4 males, 11 females) were untreated.

**Table 1:** Demographic and clinical characteristics of patients

	FD patients		Healthy control
	ERT	Non-ERT	
Gender (male, %)	7, 46.67%	4, 26.67%	13, 43.33%
Age (years)	*36.33 ± 15.12*	*39.67 ± 14.00*	*40.43 ± 16.22*
BCVA (LogMAR)	0.0 (0.0-0.1)	0.0 (0.0–0.0)	0.0 (0.0–0.0)
MSSI	11 (7, 20)	8 (2, 18)	/
α-Gal A activity (nmol/hr/ml)	1.07 (0.30, 2.19)	1.29 (0.78, 2.50)	/
lyso-Gb3 (ng/mL)	12.87 (2.79, 59.83)	2.34 (1.16, 5.54)	/
CV opacities (n, %)	11, 73.33%	7, 46.67%	/
Conjunctival abnormalities (n, %)	7, 46.67%	6, 40.00%	/
Posterior lens opacity (n, %)	4, 26.67%	5, 33.33%	/
Retinal vessel tortuosity (n, %)	6, 40.00%	5, 33.33%	/

FD: Fabry disease; ERT: enzyme replacement therapy; BCVA: best-corrected visual acuity; MSSI: Mainz Severity Score Index; α-Gal A:α-galactosidase A; lyso-Gb3:globotriaosylsphingosine; CV: corneal verticillata; Normal plasma α-Gal A activity: 2.40-17.65nmol/hr/ml; Normal plasma lyso-Gb3: <1.11 ng/ml

No significant difference was found in the mean age between FD patients and healthy controls [(38.00 ± 14.41) years vs. (40.43 ± 16.22) years, *P* = 0.541]. All patients demonstrated a median logMAR best-corrected visual acuity (BCVA) of 0.0, with no significant variations in BCVA or intraocular pressure (IOP) observed. Similarly, the mean age was comparable between male and female patients (38.00 ± 14.53 years vs. 38.00 ± 14.74 years, *P* = 1.000), as were other baseline parameters such as BCVA and IOP. However, MSSI scores were significantly different between the ERT and non-ERT patients, as were α-Gal A activity and plasma lyso-Gb3 levels. Irrespective of ERT status, older patients had higher MSSI scores than their younger counterparts (*P* = 0.001), and males had notably higher MSSI scores [median 18.00 (11.00, 25.00)] compared to females [median 7.00 (2.00, 15.00)] (*P* = 0.008). Moreover, α-Gal A activity was negatively correlated with MSSI scores (*P* = 0.001), whereas plasma lyso-Gb3 levels were positively associated with MSSI scores across all FD patients (*P* = 0.002).

**Table 2:** Baseline demographic and treatment characteristics of FD patients on ERT

Patient ID	Gender	Age	Genotype	Plasma α-Gal A activity (nmol/hr/ml)	lyso-Gb3 (ng/mL)	ERT	Dose
NO.1	F	55	c.395G > A (p.G132E)	1.66	2.78	agalsidase alfa	0.2 mg/kg
NO.2	M	39	c.1151T > A (p.1384 N)	0.30	61.07	agalsidase alfa	0.2 mg/kg
NO.3	M	10	c.1072 107 4delGAG (p.Glu358del)	0.33	43.13	agalsidase alfa	0.2 mg/kg
NO.4	F	53	c.1013 A > G(Glu338Gly)	2.17	1.73	agalsidase alfa	0.2 mg/kg
NO.5	M	18	c.395G > A (p.G132E)	0.19	59.83	agalsidase alfa	0.2 mg/kg
NO.6	F	28	c.395G > A (p.G132E)	1.63	2.82	agalsidase alfa	0.2 mg/kg
NO.7	F	57	c.695T > G(p.Ile232Ser)	10.3	4.26	agalsidase alfa	0.2 mg/kg
NO.8	M	38	c.1080_1082delTGG (p. Gly361del)	0.29	90.82	agalsidase alfa	0.2 mg/kg
NO.9	F	44	c.1072 107 4delGAG (p.Glu358del)	1.07	12.87	agalsidase alfa	0.2 mg/kg
NO.10	M	50	c.1020G-A(p. Trp340*)	0.35	92.54	agalsidase alfa	0.2 mg/kg
NO.11	F	14	c.769G > C	2.19	2.8	agalsidase alfa	0.2 mg/kg
NO.12	M	32	c.695T > G(p.Ile232Ser)	0.2	44.79	agalsidase alfa	0.2 mg/kg
NO.13	F	27	c.1013 A > G(Glu338Gly)	4.59	1.16	agalsidase alfa	0.2 mg/kg
NO.14	F	49	c.1066 C > T(p.Arg356Trp)	2.2	2.79	agalsidase alfa	0.2 mg/kg
NO.15	M	31	c.463G > A (p. Asp155Asn)	0.5	53.53	agalsidase alfa	0.2 mg/kg

FD: Fabry disease; ERT: enzyme replacement therapy; α-Gal A:α-galactosidase A; lyso-Gb3: globotriaosylsphingosine

In our investigation, ocular manifestations among FD patients included CV opacities in 18 patients, conjunctival vessel abnormalities in 13, posterior capsular lens opacities in 9, and retinal vessel tortuosity in 11. These ocular features were distributed randomly among FD patients, with no significant differences observed between those receiving ERT and those not on ERT. Notably, a significant positive correlation was found between posterior lens opacities and the severity of FD, as measured by the MSSI (χ² = 7.341, *P* = 0.019).

### Corneal epithelial morphology

High-reflective intracellular inclusions, characterized as granular cytoplasmic deposits, were detected in the basal epithelial cells of the cornea in 22 FD patients using IVCM, irrespective of ERT status. In contrast, these inclusions were not observed in the healthy controls. Notably, FD patients with CV opacities (16 out of 18) exhibited a significantly higher prevalence of these high-reflective intracellular inclusions within the corneal epithelial layer compared to those without opacities (6 out of 12).

**Fig. 1:**
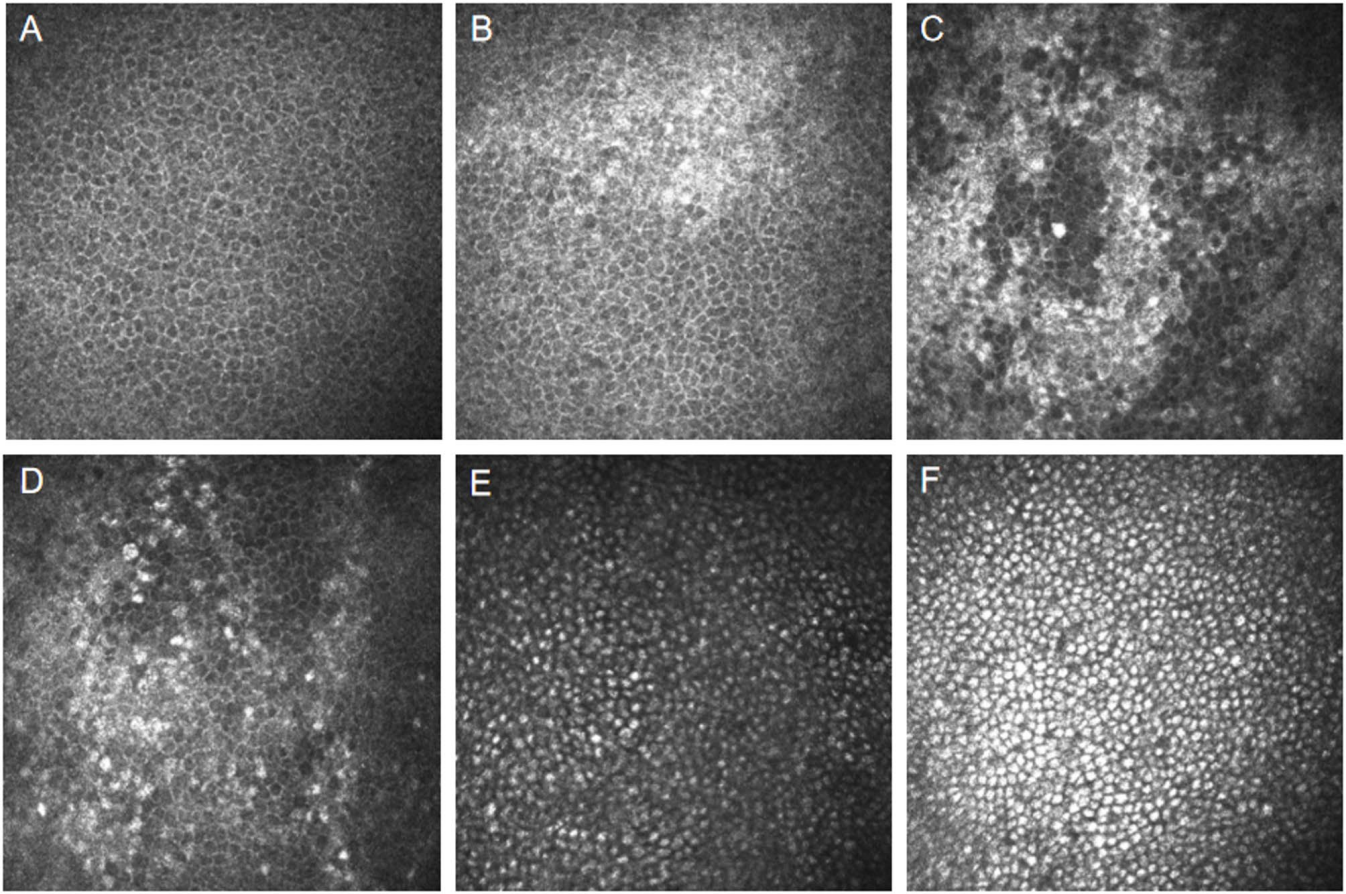
IVCM images of corneal epithelium. **A**: Normal corneal epithelium in a healthy control. **B**-**D**: Corneal epithelium from three female FD patients: **B**-**C**: Non-ERT treated. **D**: ERT-treated. **E**-**F**: Corneal epithelium from two male FD patients: **E**: Non-ERT treated. **F**: ERT-treated

In this cohort, 13 female patients (5 on ERT, 8 not on ERT) showed an irregular distribution of basal epithelial cells with interspersed high-reflective regions among normally low-reflective cells. Conversely, 9 male patients (6 on ERT, 3 not on ERT) exhibited uniformly distributed high-reflective intracellular inclusions in the corneal basal epithelial cells (Fig. 1). No significant differences were observed between the ERT and non-ERT groups. Fisher’s exact test revealed no significant differences related to ERT in the occurrence of high-reflective intracellular inclusions (*P* = 1.00), nor were there any significant gender-based differences (*P* = 0.672).

Significant increases in the area, cell density, average size, and light density of high-reflective regions in the basal epithelial cells were observed in FD patients compared to healthy controls, as analyzed using Image J software (all *P* < 0.001). However, the circularity of these high-reflective cells was significantly reduced in FD patients (*P* < 0.001) (Table 3).

**Table 3:** IVCM analysis of regions with High-Reflective intracellular inclusions in basal epithelial cells: healthy vs. FD patients

High-reflective regions in basal epithelial cells in IVCM	Healthy control	FD patients	*P* value
Area (µm^2^)	68.90 (27.67- 176.87)	1245.66 (203.72-2066.51)	<0.001
Cell density (cell number/mm^2^)	162.50 (79.69- 303.91)	795.31 (262.50-1570.31)	<0.001
High-reflective cell circularity	0.94 (0.92–0.96)	0.87 (0.85–0.90)	<0.001
Average cell size (µm^2^)	2.96 (2.41–3.79)	8.03 (4.59–11.57)	<0.001
Integrated density	698.48 (566.81- 889.76)	1886.81 (1078.60-2718.21)	<0.001

IVCM: in vivo confocal microscopy; FD: Fabry disease

### Nerve morphology

**Fig. 2:**
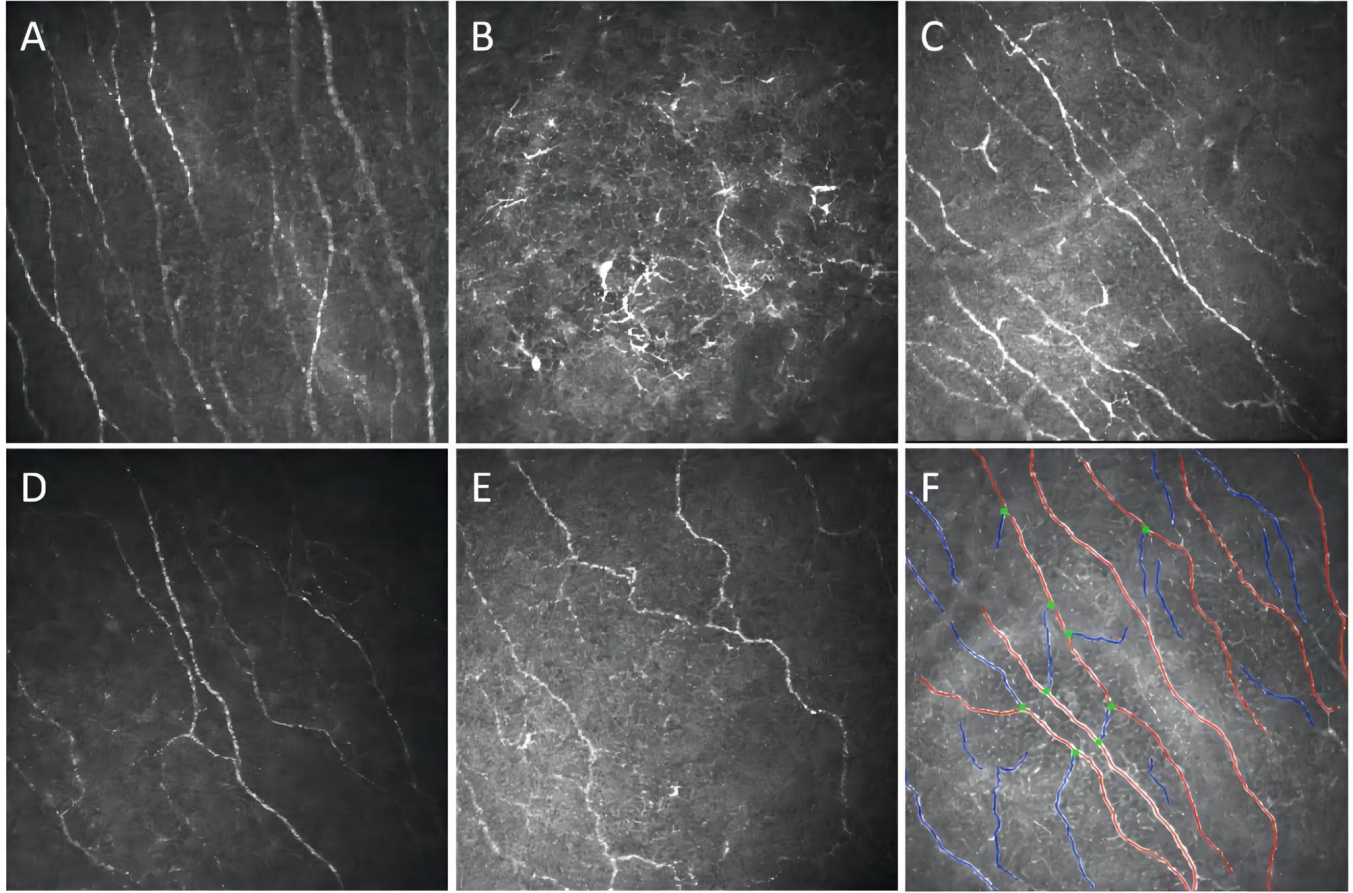
IVCM images of corneal nerve fibers. **A**: Normal corneal nerve fibers in a healthy individual. **B**-**C**: Increased dendritic cells in the nerve fiber layer in FD patients. **D**: Reduced nerve fiber density in FD patients. **E**: Increased nerve fiber tortuosity in FD patients. **F**: Quantitative analysis of neural parameters using image analysis software

IVCM findings in FD patients, both on and off ERT, revealed increased dendritic cells (DCs) in both the epithelial and nerve fiber layers, accompanied by nerve fiber tortuosity and reduced NFD (Fig. 2). Quantitative analysis revealed significant reductions in NFD (*P* = 0.033), nerve fiber length (NFL) (*P* = 0.012), and nerve fractal dimension (*P* = 0.010) in FD patients compared to healthy controls. Additionally, a significant increase in the corneal nerve tortuosity coefficient was noted (*P* < 0.001). Despite these morphological changes, no significant differences were observed in these parameters within the nerve fiber layers between patients receiving and not receiving ERT (Table 4).

**Table 4:** IVCM analysis of nerve fiber layer: healthy individuals vs. FD patients and ERT-Treated vs. Non-ERT patients

Nerve fiber layer in IVCM	Healthy control	FD patients	*P* value	FD on ERT	FD not on ERT	*P*’ value
NFD (fibers/mm²)	18.75 (12.50-19.53)	12.50 (9.37–18.75)	0.033	15.21 ± 12.38	14.37 ± 4.84	0.811
NBD (branches/mm²)	17.19 (8.59–28.90)	12.50 (3.12-25.00)	0.091	14.58 ± 11.18	13.54 ± 10.8	0.797
NFL (mm/mm²)	13.71 ± 3.28	11.32 ± 3.79	0.012	11.38 ± 4.75	11.26 ± 2.68	0.932
NFA (10^− 3^mm^2^)	5.98 ± 1.55	5.63 ± 1.57	0.389	5.58 ± 1.66	5.69 ± 1.53	0.852
NFW (10^− 2^mm)	2.22 (2.10–2.30)	2.18 (2.10–2.29)	0.882	2.13 (2.10–2.28)	2.24 (2.13–2.37)	0.116
fractal dimension	1.48 ± 0.03	1.45 ± 0.05	0.01	1.45 ± 0.05	1.45 ± 0.04	0.824
tortuosity coefficient	11.95 ± 4.06	20.70 ± 6.17	<0.001	19.97 ± 5.08	21.42 ± 7.20	0.529

P’ means P value between ERT group and non-ERT group

IVCM: in vivo confocal microscopy; FD: Fabry disease; ERT: enzyme replacement therapy; NFD: nerve fiber density; NBD: nerve branch density; NFL: nerve fiber length; NFA: nerve fiber area; NFW: nerve fiber width

### Corneal stromal cell morphology

**Fig. 3:**
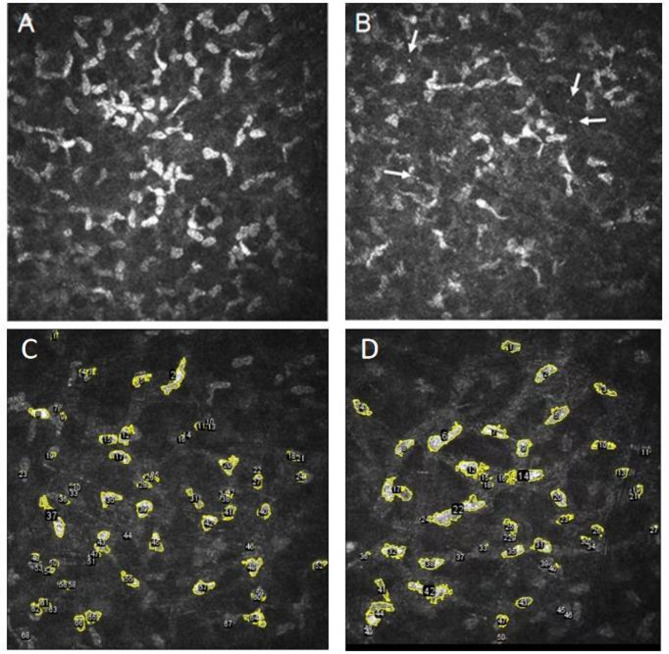
IVCM of corneal stroma. **A**-**B**: Corneal stroma in FD patients. Microdots indicated by white arrows. **C**: Cell borders in a healthy control. **D**: Cell borders in a FD patient

Among thirteen FD patients, seven receiving ERT and six not on ERT, IVCM revealed decreased transparency of the anterior corneal stroma characterized by visible microdots (Fig. 3). Quantitative analysis indicated no significant differences in total area (8586.48 ± 5125.67 vs. 10213.2 ± 4648.15, *P* = 0.203), cell density (412.5(308.59,443.75) vs. 387.5(313.28,453.91), *P* = 0.894), average cell size (109.89(93.52,171.44) vs. 149.15(119.53,191.33), *P* = 0.065), cell circularity (0.45 ± 0.04 vs. 0.45 ± 0.03, *P* = 0.495), or integrated stromal density (25825.91(21977.42,40290.71) vs. 35050.34(28090.86,44964.44), *P* = 0.065) between FD patients and healthy controls. Similarly, comparisons between the ERT and non-ERT groups showed no significant differences in these parameters (total area: *P* = 0.481, cell density: *P* = 0.693, average cell size: *P* = 0.272, cell circularity: *P* = 0.794, integrated stromal density: *P* = 0.272).

### Endothelial morphology

Endothelial morphological parameters in FD patients showed no significant differences compared to healthy controls. Parameters analyzed included endothelial cell density (1754.45 ± 271.08 vs. 1780.39 ± 346.01, *P* = 0.748), cell size (473.13 ± 80.04 vs. 441.94 ± 93.95, *P* = 0.172), Feret diameter (28.52 ± 2.56 vs. 28.21 ± 2.36, *P* = 0.631), minimum Feret diameter (22.81 ± 1.98 vs. 22.42 ± 1.69, *P* = 0.414), and endothelial integrated density (79537.17 ± 22081.02 vs. 71361.32 ± 17359.25, *P* = 0.116). Additionally, comparisons between patients undergoing ERT and those not on ERT revealed no significant differences in cell density (*P* = 0.933), average cell size (*P* = 0.669), Feret diameter (*P* = 0.634), minimum Feret diameter (*P* = 0.542), and endothelial integrated density (*P* = 0.167).

## Discussion

FD, being a rare condition, presents with a broad spectrum of clinical manifestations, many of which are non-specific and can often lead to misdiagnosis, particularly in individuals without a family history or with atypical symptoms. The diagnostic complexity is further exacerbated by the variability in clinical presentation, necessitating a high index of suspicion for timely detection [17, 18]. Early diagnosis is crucial, as the initiation of ERT can lead to significant improvements in disease progression, especially when started early [8, 19–22]. However, in mainland China, the adoption of ERT is influenced not only by clinical indications but also by socio-economic factors, including the patient’s financial capacity and willingness to undergo treatment [23, 24].

Ocular lesions in FD are generally non-vision-threatening but serve as distinct early markers, identifiable via routine, non-invasive ocular examinations [1, 9]. Common ocular findings include CV opacities, which arise from the accumulation of glycosphingolipids within the corneal epithelium, typically near or at Bowman’s membrane. In the Fabry Outcome Survey, CV opacities were observed in 76.9% of females and 73.1% of males with FD [9, 25]. In contrast, our cohort presented with a slightly lower incidence: 52.6% of females and 72.7% of males. Interestingly, the incidence was higher in ERT-treated patients (73.33%) compared to those untreated (46.67%). However, MSSI scores did not differ significantly between those with and without CV opacities, reinforcing previous findings that these opacities do not correlate directly with the overall severity of FD or systemic involvement [9, 26–28].

We employed IVCM for detailed assessment of corneal epithelium, nerve fiber layer, stroma, and endothelium in various cohorts. In line with findings by Leonardi et al., our analysis identified high-reflective intracellular inclusions within the basal epithelial cells, an elevated DCs count, and increased nerve fiber tortuosity in the nerve fiber layer of FD patients. Furthermore, we noted a reduction in NFD, NFL, and nerve fractal dimension, alongside reduced anterior corneal stromal transparency and the occurrence of microdots, regardless of ERT status [28–30].

Consistent with Mastropasqua et al., our study identified distinctive morphological features of high-reflective intracellular inclusions in the basal epithelium of FD patients. Male patients showed well-defined, round intracellular inclusions uniformly distributed within the basal epithelial cell layer, whereas female patients exhibited a fragmented reflective pattern termed ‘fine diffusion’ throughout the epithelial layers, deviating from typical female patterns [25]. Additionally, those with CV opacities exhibited a higher frequency of inclusions compared to those without CV opacities [30]. Notably, even in the absence of CV opacities, half of the FD patients displayed these intracellular inclusions, suggesting that IVCM may be a more sensitive diagnostic tool than traditional slit-lamp examination for detecting FD. It is also important to note that similar high-reflective intracellular inclusions and CV opacities are seen in amiodarone users, which underscores the need to differentiate between FD and other conditions with similar ocular manifestations [29, 31].

Our findings indicate a notable rise in DCs count in FD groups. DCs are implicated in migrating and accumulating in the central cornea during various neuropathies and inflammatory conditions [32]. Elevated levels of inflammatory cytokines, including interleukin-6 (IL-6) and tumor necrosis factor-alpha (TNF-α) in FD, indicate an inflammatory state and suggest a possible autoimmune component in its pathophysiology [33]. Interestingly, our study revealed that the DCs count was significantly elevated not only in the corneal sub-basal nerve plexus but also in the epithelial layer, contrary to some previous reports [28]. This discrepancy could be attributed to factors such as small sample size or potential racial differences in disease presentation.

Our observations of reduced NFD, NFL, and nerve fractal dimension, along with increased nerve fiber tortuosity, are consistent with previous studies that report corneal neuropathy in FD, which may also correlate with systemic small fiber neuropathy [1]. In conjunction with reduced corneal sensitivity, these findings suggest that FD could contribute to dry eye disease in affected individuals, as noted in previous literature [1, 34, 35].

Similar to the findings of Wasielica-Poslednik J et al., this study identified decreased transparency in the anterior corneal stroma and detected microdots in specific FD patients [29]. Similarly, Libert et al. reported the presence of anterior stromal microdots in FD patients, attributing these to lipid deposits [36]. Nonetheless, it should be emphasized that the presence of microdots and decreased stromal transparency are non-specific findings. These phenomena have been documented not only in FD patients but also in individuals with amiodarone-induced keratopathy and even in asymptomatic elderly populations [29].

No significant differences in corneal endothelial morphology were observed between the FD cohort and healthy controls, consistent with previous studies [28, 30, 37]. This raises the possibility that FD may not significantly affect the corneal endothelium or that subtle changes were missed due to the limitations of IVCM, which may not be sensitive enough to detect early endothelial alterations. Further studies with advanced imaging techniques are needed to clarify any potential endothelial involvement in FD.

In our cohort, α-Gal A activity inversely correlated with MSSI scores, while plasma lyso-Gb3 levels exhibited a direct correlation with MSSI scores, consistent with previous research [38–40]. The novel correlation observed between the severity of posterior capsular opacity and MSSI (χ² = 7.341, *P* = 0.019) suggests that lenticular changes could serve as a potential marker for disease progression. When combined with lyso-Gb3 levels, this creates a dual-modality monitoring framework: Lyso-Gb3 as a molecular indicator of disease burden and lens grading as an accessible structural marker. This approach could optimize surveillance, especially in regions where advanced imaging modalities are not readily available.

Our study is constrained by several limitations. First, the small sample size and exclusive recruitment from cardiology referrals may introduce selection bias, potentially skewing the spectrum of clinical phenotypes and FD subtypes. Second, the descriptive study design precludes longitudinal tracking of ocular symptoms and signs across the natural progression of FD, failing to document changes in ocular manifestations pre- versus post-ERT. Third, the absence of standardized ERT regimen documentation—including cumulative dosage and treatment duration—limits the ability to establish dose-response relationships with ocular outcomes. Additionally, the lack of corneal sensitivity and visual quality assessments also restricts the comprehensiveness of our conclusions. Future prospective studies should address these limitations by incorporating longitudinal assessments, standardized ERT protocols, and more comprehensive evaluations of visual function.

## Conclusions

This study provides valuable insights into FD-related ocular alterations in the mainland Chinese population. Ophthalmic evaluation proves to be a crucial clinical tool for early FD screening, especially when combined with newborn screening or family history assessments [41]. Beyond facilitating early diagnosis, specific ocular findings—such as CV opacities, posterior capsular opacification, and distinctive IVCM changes—can serve as potential biomarkers for FD at early stages. These markers should prompt further investigation into the patient’s clinical symptoms and family history to confirm the diagnosis. Early identification of FD through ophthalmic markers can lead to timely intervention and more effective management.

## Data Availability

The data are available on request. The data are not publicly available due to privacy restrictions. Data requests can be made to this email: zhangxiaofeng@suda.edu.cn.

## Abbreviations

FD: Fabry disease
α-Gal A: α-galactosidase A
GL-3: Glycosphingolipid globotriaosylceramide
lyso-Gb3: Globotriaosylsphingosine
ERT: Enzyme replacement therapy
CV: Corneal verticillate
IVCM: In vivo confocal microscopy
MSSI: Mainz Severity Score Index
NFD: Nerve fiber density
NBD: Nerve branch density
NFL: Nerve fiber length
NFA: Nerve fiber area
NFW: Nerve fiber width
BCVA: Best-corrected visual acuity
IOP: Intraocular pressure
DCs: Dendritic cells
IL-6: Interleukin-6
TNF-α: Tumor necrosis factor-alpha
